# Perception of COVID-19 Testing in the Entire Population

**DOI:** 10.3389/fpubh.2022.757065

**Published:** 2022-02-10

**Authors:** Beata Gavurova, Viera Ivankova, Martin Rigelsky, Zdenek Caha, Tawfik Mudarri

**Affiliations:** ^1^Institute of Earth Resources, Faculty of Mining, Ecology, Process Control and Geotechnologies, Technical University of Košice, Košice, Slovakia; ^2^Department of Marketing and International Trade, Faculty of Management and Business, University of Prešov, Prešov, Slovakia; ^3^Department of Human Resource Management, Faculty of Corporate Strategy, Institute of Technology and Business in České Budějovice, České Budějovice, Czechia

**Keywords:** pandemic, political decision-making, interventions, mass testing, behavior, gender, age, social attitudes

## Abstract

In the Slovak Republic, a mass testing of the entire population was performed. Estimates show that this testing cost more than 400 million EUR and thousands of euros were paid for one positively identified case. Thus, it is possible to state a high cost for such a project, which has been criticized by many parties. On the other hand, from a public health point of view, mass testing has helped fight the pandemic. Both the health and economic perspectives are important in assessing the success of a pandemic strategy, but the social perspective is equally important. In fact, the situation is perceived from the position of public leaders who make decisions, but also from the position of the society that bears individual political decisions. It is not appropriate to forget about the society that is most affected by restrictions, testing, health status, but also the burden on the state budget. The objective of the presented research was to examine the perception of testing for coronavirus disease 2019 (COVID-19) in the Slovak population. Non-parametric difference tests and correspondence analysis were used for statistical processing. The research sample consisted of 806 respondents and data collection took place in February 2021. The main findings include significant differences in perceptions between the first and the last participation in testing in terms of gender, age, testing experience, and time aspect. The last participation in testing showed lower rates of positive aspects related to the internal motivation to test compared to the first participation. In contrast, external stimulation by government regulations related to restrictions in the absence of a negative result was higher in the last participation in testing. There were also differences between the first and the last test in the level of doubts about the accuracy of the test result, while a higher level was found at the last testing participation. It can be concluded that the frequency of testing and its requirements need to be approached very carefully over time, as it is likely that the positive perceptions may deteriorate. The recommendations include clear and timely government communication, trust building and health education.

## Introduction

Great attention was paid to the testing strategy in the Slovak Republic. This leader's decision concerned the country's public health and national economy. In the Slovak Republic, as the first country in Europe, antigen tests have been used in the entire population, contrary to the recommendations of experts in mass testing. As the government adopted a strategy for mass testing of the entire population, testing was also conducted in regions with a low incidence of infection. From an economic point of view, internal estimates showed that mass testing by April 2021 amounted to more than 400 million EUR, and the efficiency was estimated at 3 000 EUR per positive case confirmed by antigen test in April 2021. The Slovak Republic started paying more than a thousand EUR for finding one infected person using antigen tests in March 2021. On the other hand, from a public health point of view, mass testing could help fight the pandemic (1). According to Frnda and Durica (2), mass testing only slowed down the disease spreading by 3–4 weeks. After a month, the daily positive cases were at the same level as before mass testing. In addition to the economic and health aspects, it is necessary to take into account social aspects. The situation is perceived from the position of public leaders who make decisions, but also from the position of the society that bears individual political decisions. It is not appropriate to forget about the society that is most affected by restrictions, testing, health status, but also the burden on the state budget. For these reasons, this study focuses on the social dimension of the problem.

Since the end of summer 2020, an increase in the number of COVID-19 cases has been evident in the Slovak Republic. The events and circumstances in the country have already been described several times (1, 3–5). During the autumn of 2020, thousands of new infected individuals were daily identified, with the share of positive tests ranging from 10.48% (4 October 2020) to 19.31% (22 October 2020) (6). Hospitals were in danger of collapsing and it was decided to carry out mass testing. The pilot round took place in four selected counties from 23 to 25 October 2020, while the first round of mass testing took place between 31 October and 1 November 2020, followed by the second round in selected counties with a high prevalence of the disease (between 7 and 8 November 2020). The testing was voluntary, but anyone who did not participate must be isolated for ten days. Another round of testing for COVID-19 took place in 458 cities and municipalities. These were cities and municipalities where the share of positive tests was 1% and more. Thereafter, voluntary testing and suspicion testing were applied during December 2020 and January 2021. The situation in this small country was serious. Based on data from the Slovak National Health Information Center (6), the 7-day average positivity rate was 27.41% and the 7-day average mortality represented almost 92 deaths from COVID-19 (from 28 December 2020 to 3 January 2021). Subsequently, a decision was made on further population-wide screening, which took place from 18 to 25 January 2021, while the further round of screening took place in the most affected counties from 27 January to 2 February 2021. After screening, cross-population testing continued on a regular basis when the “COVID automat” was launched in the country (8 February 2021) (7).

In the vast majority of cases, rapid antigen tests were used for the early identification of SARS-CoV-2 infected individuals during mass testing, screening and regular monitoring of the Slovak population (6). A negative test result was required, for example, on the way to work, accompanying a child to kindergarten or pupil and student to school; on the way to a dry cleaner, optics, bank, insurance company, bicycle and motor vehicle service, post office, technical and emission control services for vehicles, shoe repair, telecommunication service, e-shop dispensing point; stay in nature, including individual sports outside the county, and others (7). In accordance with the regulations, random checks were carried out and fines were imposed for infringements. At this point, it should be noted that in addition to testing, other interventions were implemented and gradually tightened (curfew, home office), which subsequently limited social contact and mobility in order to reduce the incidence of COVID-19. There was also a lockdown that could restrict “looser” contacts, leading to obscure transmissions that are difficult to identify.

With regard to Standard Q rapid antigen tests used in most mass testing in the Slovak Republic, there were evidence showing that 6.8% of patients with a suspicion of SARS-CoV-2 infection were false-negative (8). A possible problem of false-negative results may be low viral load along with low viable virus and low infectiousness (9), but also lower sensitivity of antigen tests compared to real-time reverse transcription polymerase chain reaction (RT-PCR) tests (10). The information sheet of Standard Q rapid antigen test states that “a negative result may occur if the concentration of antigen in a specimen is below the detection limit of the test or if the specimen was collected or transported improperly, therefore a negative test result does not eliminate the possibility of SARS-CoV-2 infection, and should be confirmed by viral culture or molecular assay” (11). Simultaneously, the sensitivity of antigen tests for detecting specimens from COVID-19 patients ranged from 58.1 to 75.9% (8–10, 12). In a Slovak study (2), the authors estimated that antigen tests positivity during the mass testing oscillated between 40 and 76% (combination of antigen tests of three companies). It should also be noted that these validations of antigen tests were performed in patients with symptoms such as fever, anosmia, sore throat, myalgia, cough and headache, and this type of tests was recommended during the first 5 days of disease, as the sensitivity and accuracy of the tests were lower after this period (10). The authors of other studies also recommended the use of rapid antigen tests within seven days after the onset of symptoms (12–14). The above-mentioned findings suggested that antigen testing makes sense especially in outbreaks with a high viral load and in people with early symptoms, when rapid antigen tests represent a COVID-19 filter. Thus, RT-PCR tests remain the reference method for detecting SARS-CoV-2 infection (15, 16).

In any case, testing as such is considered a key contribution to the successful management of the COVID-19 pandemic, differing only in its form, organization and regime. This fact was supported by findings from a study focused on the effect of population-wide rapid antigen testing on the prevalence of SARS-CoV-2 in the Slovak Republic (1). The authors concluded that the combination of national restrictions (including contact reduction measures) and mass testing together with the requirement for quarantine for the whole household reduced the prevalence of infected persons (1). It is up to countries to decide which effective strategies and programs to choose for the early diagnosis of the viral infection and to prevent its spread in the population. According to several authors, rapid antigen tests and RT-PCR tests are comparable tools for the detection of SARS-CoV-2 (17), and mass testing can be one of effective public health tools in combination with various interventions (1). The approach to detecting infection by testing symptomatic people and their contacts can be a slow and weak approach in preventing the spread of the virus in the community. Thus, attention should also be paid on asymptomatic people, as they may contribute to the transmission of SARS-CoV-2. Rapid antigen tests have shown sufficient sensitivity in detecting cases of infection with a higher viral load, especially in regions with a high prevalence and in environments where it is not possible to maintain physical distance (e.g., at work and at school) (18). Mass testing may help to control pandemics, but it should be borne in mind that much evidence has supported the focus of testing on areas, populations or places with higher incidence and risk, while other interventions should not be forgotten (19).

The social and behavioral responses to mass asymptomatic testing in the population are not fully understood. International studies focused on barriers and concerns regarding testing in various countries around the world, but the testing strategy in the Slovak Republic was different. Nevertheless, the common characteristic can be recognized in many countries. It has been found that when people perceive the stigma associated with COVID-19, they have low confidence in health institutions and doubts about the procedural integrity of the testing process. In other words, the willingness to participate in testing is influenced by stigma, trust in institutions and expectations regarding the integrity of tests (20). Also, the main general obstacles and concerns included confusion and uncertainty about testing instructions and where to go for testing, lack of available testing places, perception that the nasal swab method is too painful, and long waiting times for results (21).

The above-mentioned facts could also be reflected in people's attitudes toward their participation in testing, as there was a heated debate in society about the conditions, organization, implementation and effectiveness of testing. The population-wide testing was the focus of attention not only of scientists, experts, the media, but also the general public, while different groups had different views. The accuracy of test results in the general population was also a question, while in many cases people had no symptoms but their participation was encouraged. This was the motivation for conducting this study focused on the social aspect of the perception of testing in the Slovak population. At the same time, it was necessary to think about people's behavior after testing, as a negative test result allowed more opportunities for mobility. It was a kind of “ticket to a freer life” alongside pandemic measures compared to individuals who did not participate in testing. Regarding the novelty, the study captures the unique situation in the Slovak Republic during the COVID-19 pandemic and provides valuable findings in this difficult situation for the public as well as for policy-makers. Mass testing, as it was in the Slovak Republic, did not occur in other countries. This study provides important findings that are useful for public health leaders in managing the pandemic with respect to the social aspect and the willingness of people to test. In this context, the study brings a new view on testing policy from a society perspective. It should be noted that this problem has not been sufficiently examined in the research area, which could be an obstacle to evidence-based interventions.

## Materials and Methods

### Research Objective and Measures

The objective of the presented research was to examine the perception of COVID-19 testing in the Slovak population. This was achieved by comparing the testing-related attitudes identified in both the first and the last participation in COVID-19 testing.

The attitudes were surveyed on several levels. At the first level, five questionnaire items identified an internal motivation to participate in testing, and one questionnaire item identified an external stimulation due to restrictions when not participating in the COVID-19 testing. The individual items of the questionnaire were measured on a 4-point scale with numerical coding from 1 to 4 (1–definitely no, 2–rather no, 3–rather yes, 4–definitely yes). All data on these attitudes were collected during the same period (from 12 February to 23 February 2021). In other words, the attitudes toward the first participation in testing were collected during the same period as for the last participation in testing. The questionnaire was divided into two parts, i.e., one for the first participation in testing and one part for the last participation in testing. The time difference between the first and the last participation was also measured. Most of the first participations could be attributed to the first mass testing conducted in the country, i.e., between 31 October and 1 November 2020. As this was a unique situation that left an emotional mark in society, participants were able to remember and adequately express their attitudes. For all of the following items, respondents provided their attitudes in terms of the first and the last participation in testing. Thus, all of the following items were requested in the part on first participation as well as in the part on last participation:

- Q_1: Did you feel that you were doing something right when participated in the testing?
- Q_2: When you participated in the testing, did you feel that the testing was beneficial?
- Q_3: Did you feel that it helped the society when you participated in the testing?
- Q_4: Did your participation in the testing make you feel safe (regardless of the test result)?
- Q_5: Did the reasons for your participation in the testing also include responsibility for the people you meet?
- Q_6: You have participated in the testing to avoid the restrictions imposed by government regulations.

Subsequently, the level of perception of doubts about the accuracy of the test result was determined on a numerical coding scale from 1 to 5. The note was given only for limit values (1–I was not at all aware, 5–I was fully aware).

- Q_7: To what extent have you realized that the test result may not be accurate?

It was also focused on how respondents spent time after testing in terms of interacting with other people. For this purpose, a questionnaire item was provided, in which respondents could choose one of the following options: (i) I went home, (ii) I met people while I knew that everyone had a negative test result, (iii) I met people while I knew that someone of them has a positive test result, (iv) I met people while I did not have information about their test result (taking into account their health status in relation to coronavirus).

- Q_8: How did you spend time after your testing in terms of meeting other people?

Subsequently, the primary reason for the meeting was identified by a questionnaire item, in which respondents could choose one of the following options: (i) I have not met anyone, (ii) work, (iii) family reunion, (iv) sports, (v) entertainment.

- Q_9: What was the purpose of the meeting after testing?

At this point, it should be noted that the survey's efforts also focused on whether participation in testing took place during population-wide testing for coronavirus in Slovakia. In this sense, it can be concluded that the first testing was carried out predominantly during the round of population-wide rapid antigen testing (76.7%). This ratio was lower at the last participation in testing, as 52.4% of respondents stated that they had been tested during another round of population-wide rapid antigen testing.

The collection of data was completed in 12 days, namely from 12 February 2021 to 23 February 2021. The collection process can be characterized as quota sampling (by gender characteristics, age and social status), which took place in two forms. The first form of collection was the paid promotion of the questionnaire with a controlled targeting of the audience on a social network. The second form of collection consisted of sending messages and e-mails requesting the completion of the questionnaire, as well as distributing and sharing the questionnaire in various groups on the social network.

### Respondents and Procedure

A total of 958 statistical units (respondents) were collected. The final research sample consisted of 806 respondents. The exclusion of the respondents' answers was as follows: firstly, the answers of respondents who did not approve their content and did not agree with the processing of their data (*n* = 42; 4.38%), subsequently, the answers of respondents who did not participate in any of the testing (*n* = 93; 9.71%), then the answers of respondents with a system error in recording their answers (*n* = 2; 0.21%), then the answers of respondents living outside Slovakia (*n* = 12; 1.25%), and, finally, the answers of respondents who were < 18 years old (*n* = 3; 0.31%). The respondents whose responses were considered irrelevant (due to the doubts they stated in open item, or a logical error) were included in the set of excluded statistical units described above. These adjustments excluded a total of 152 statistical units.

At the beginning of the questionnaire, all respondents received the same information about the research and they were provided with information about their rights and anonymity. All respondents included in the presented research confirmed their informed consent. The respondents were reminded that there were no correct or incorrect answers, only their actual attitudes, and they were asked to complete the questionnaire responsibly. The respondents did not receive any financial reward. All aspects in this research were conducted with respect to the seventh revision of the World Medical Association–Declaration of Helsinki.

Table 1 shows the frequencies of the selected identifiers in the research sample. It is clear that there was a certain predominance of females and students (i.e., younger respondents), which was due to the low willingness of males to fill in the questionnaire and, conversely, the high willingness of females as well as students in general. The age of the respondents (year of birth) was identified by an open questionnaire item, while the table already provides the categories determined by the decade. This made it possible to present generational differences. The histogram of individual age categories is provided in Supplementary Figure 1. The average year of birth of the respondent was 1989 (median: 1995), which means an average age of 32 years, with the oldest respondent being born in 1950 (71 years) and the youngest in 2003 (18 years). The variable determining the number of participations in testing was also collected through an open questionnaire item and subsequently adjusted according to the categories listed in Table 1. The mean number of tests was 5.65 (median: 5, mode: 4), with a minimum of 1 and a maximum of 32 tests. In six cases, the respondents did not fill in the item numerically (they stated “a lot” or another term) and these responses were considered missing values. Regarding the time between the first and the last test of the respondents, the most frequented category was “more than a month” and the least frequented category was “about 2 weeks” (at the time of data collection). Also, 32 of the 33 missing values represented only one participation in testing. One missing value was probably caused by an error on the part of the respondent who did not complete the item. There were more similar cases in the items related to the last participation in testing, but these were only rare cases counted in units.

**Table 1:** Descriptive profile of the research sample.

**Characteristics**	***N*** **(806)**	**Percent**
**Gender:**		
Males	314	39.0%
Females	492	61.0%
**Social status:**		
Full-time student	364	45.2%
Employed	317	39.3%
Entrepreneur and others	50	6.2%
Unemployed	31	3.8%
Maternity leave/guardianship	18	2.2%
Pensioner (old-age, disabled)	26	3.2%
**Year of birth:**		
<1980 (>41 years)	176	21.8%
1980–1989 (32–41 years)	113	14.0%
1990–1999 (22–31 years)	427	52.9%
2000+ (<22 years)	90	11.2%
**Number of tests:**		
≤ 5	434	53.8%
6+	366	45.4%
Missing	6	0.7%
**Time between the first and the last test:**		
About a week	68	8.4%
About two weeks	17	2.1%
About a month	29	3.6%
More than a month	659	81.8%
Missing	33	4.1%

### Statistical Approach

Non-parametric tests were used for statistical processing. These methods were preferred due to the failure to meet several assumptions for the use of parametric methods (as data in an ordinal scale were used). Differences in perception between the first and the last COVID-19 testing were assessed using the Wilcoxon signed-rank test for two dependent samples. This test was selected based on the apparent relationship between the first testing and the last testing, i.e., it was the same person. The differences across gender and age groups were determined using the Wilcoxon signed-rank test for two independent samples and the Kruskal Wallis H test for k independent samples. Correspondence analysis was chosen to assess the links between the overall perception of testing and the gender-age characteristics, while the suitability (relations of the analyzed categories of selected variables) was verified using Pearson's χ^2^ test. This analysis was considered appropriate based on the fact that the analyzed data were in nominal and ordinal scales. Analytical calculations were performed using the programming language R v 4.0.3 (RStudio, Inc., Boston, MA, USA).

## Results

The analytical processing was divided into three main parts in the context of the purpose of this study. In the first part, attention was focused on attitudes to COVID-19 testing (Q1–Q6). Thus, the differences in perceptions between the first test and the last test were pointed out in this part. In the second part, attention was focused on assessing the perception of doubts about the accuracy of the test result. As in the first part, the second part was based on a comparison of the first and the last participation in testing. In both parts, the comparison was extended to include the links between the categories of gender and age characteristics, which were presented using correspondence analysis. The third part was devoted to the behavior of respondents after testing.

### Perception of Testing

**Figure 1:**
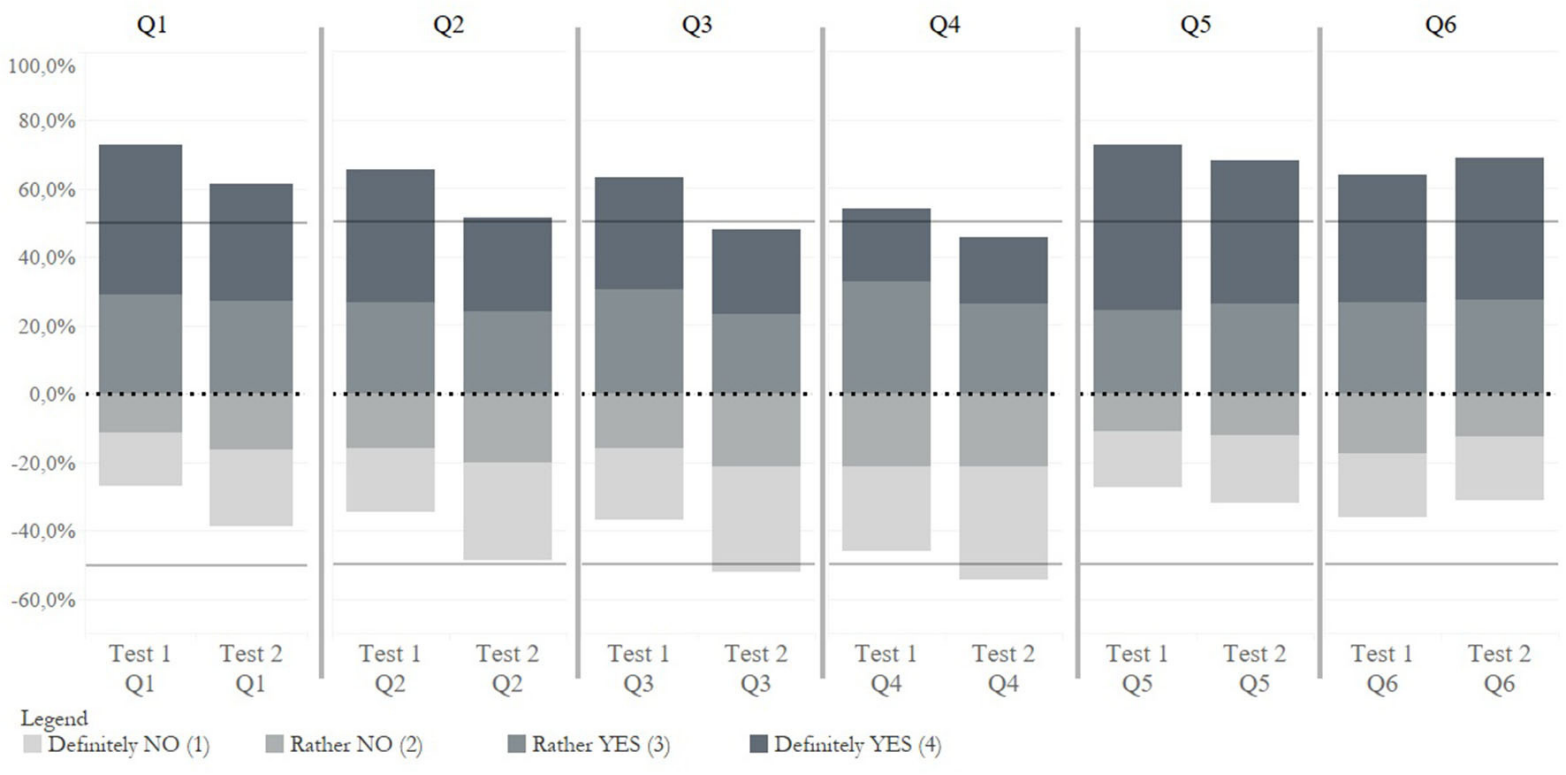
Visualization of COVID-19 testing perceptions–the first test and the last test.

Figure 1 shows the outputs of respondents' perception of testing. This figure can be seen in the light that the higher the value, the more positive the perception (Q1–Q5). It is clear that some differences were observed between the first test and the last test, indicating that the respondents' last participation in testing showed a less positive perception of testing in response to items Q1–Q5. On the contrary, higher values were observed for the last item Q6. This item aimed to identify the level of external stimulation by government regulations to encourage people to test. Otherwise, restrictions were adopted for people who did not participate in the testing. It can be stated that the last participation in the COVID-19 testing was to a greater extent conditioned by the restrictions, resulting in an increased rate of participation to avoid the restrictions imposed by government regulations. This was expected due to the gradual tightening of regulations. The fact that the last participation in testing was characterized by a loss of people's internal motivation cannot be considered positive. In other words, it was possible to observe a decline in the feeling that they had done something good for society, their loved ones or their personal sense of safety.

Table 2 shows the values of the statistical characteristics of the central tendencies, as well as the results of the difference tests (Wilcoxon signed-rank test for two dependent samples). The table provides the results without classification, the results classified by time between the first test and the last test (identification of the time aspect of testing) and the results classified by number of tests (identification of test intensity). Differences in perception between the first test and the last test without classification were significant in all items. Based on the values of the central tendencies for items Q1–Q5, the results indicated that the last participation in testing showed significantly lower values compared to the first participation. This means that the internal motivation was significantly lower at the last participation in testing, when people felt less intensely that their participation was right, beneficial, helpful, responsible to other people and evoking a sense of safety. On the contrary, a significantly higher value for the last test was shown in the context of external stimulation by government regulations, and thus in people's efforts to avoid the restrictions (Q6: You have participated in the testing to avoid the restrictions imposed by government regulations). A similar direction of values can be observed within the significant results in individual classifications, indicating that respondents perceived the last participation in testing less positively. This statement is based on the fact that respondents reported a lower positive internal motivation to be tested at the last participation in testing. In other words, the last testing was less perceived as right, beneficial, helpful, responsible and safe compared to the first testing. At the same time, in comparison with the first testing, the answers to the questionnaire item Q6 indicated that the last testing was more encouraged by the intention to avoid restrictions in public life. This interpretation is used throughout the study.

**Table 2:** COVID-19 testing perceptions–the first test and the last test in the classification of time between tests and the number of tests.

		**Q1**	**Q2**	**Q3**	**Q4**	**Q5**	**Q6**
**All (*****n*** **=** **806)**							
T1	Mean	3.00	2.86	2.75	2.51	3.05	2.82
	Median	3	3	3	3	3	3
T2	Mean	2.74	2.51	2.42	2.32	2.91	2.93
	Median	3	3	2	2	3	3
Diff	Z	−10.09^†^	−10.84^†^	−10.53^†^	−7.35^†^	−6.06^†^	−3.40^***^
**Less than a month (*****n*** **=** **114)**							
T1	Mean	2.58	2.48	2.37	2.23	2.73	2.88
	Median	3	3	2	2	3	3
T2	Mean	2.50	2.30	2.23	2.07	2.59	2.83
	Median	3	2	2	2	3	3
Diff	Z	−1.08	−2.45^**^	−1.65^*^	−2.38^**^	−1.92^*^	−0.57
**More than a month (*****n*** **=** **659)**							
T1	Mean	3.13	2.98	2.86	2.60	3.16	2.79
	Median	3	3	3	3	4	3
T2	Mean	2.78	2.55	2.45	2.36	2.96	2.95
	Median	3	3	2	2	3	3
Diff	Z	−10.19^†^	−10.57^†^	−10.56^†^	−6.95^†^	−5.77^†^	−3.77^†^
**Up to 5 tests (inclusive) (*****n*** **=** **402)**							
T1	Mean	2.80	2.64	2.56	2.35	2.89	2.97
	Median	3	3	3	2	3	3
T2	Mean	2.59	2.37	2.28	2.19	2.81	2.93
	Median	3	2	2	2	3	3
Diff	Z	−6.38^†^	−6.60^†^	−7.27^†^	−5.41^†^	−3.52^†^	−0.003
**More than 5 tests (*****n*** **=** **366)**							
T1	Mean	3.24	3.11	2.97	2.69	3.24	2.64
	Median	4	3	3	3	4	3
T2	Mean	2.59	2.37	2.28	2.19	2.81	2.93
	Median	3	2	2	2	3	3
Diff	Z	−8.21^†^	−8.95^†^	−7.76^†^	−5.04^†^	−5.25^†^	−4.83^†^

*T1, first test; T2, last test; Diff, differences*;

* *p-value < 0.1*;

** *p-value < 0.05*;

*** *p-value < 0.01*;

† *p-value < 0.001*.

With a focus on the time aspect of testing, it is clear that this aspect could have been significantly reflected in the perception of testing. When comparing the group of respondents with less than a month between testing and the group of respondents with more than a month between testing, some deviations in the significance of differences were observed. Thus, the differences in perception between the first test and the last test were more intense in the group with a longer time interval between tests. However, these results confirmed that, in most cases, the last testing was perceived significantly less positively in both time periods. Similar results were revealed in the groups classified by number of tests (up to 5 tests (inclusive), more than 5 tests). The differences in perception between the first and last test were more intense in the group with a larger number of tests. The only exception was found for Q6, in which no significant difference could be observed in the group with a smaller number of tests. Overall, the last testing was perceived significantly less positive than the first testing in both groups of respondents divided by the number of participations in testing.

Table 3 provides the values of the central tendencies, as well as differences in the perception of the first testing and the last testing across gender and age groups. The significant gender differences in the perception of the first testing were found in three items (Q3, Q4, Q5), in which females showed higher values. Thus, females perceived their first participation in testing as helpful, responsible, and evoking a sense of safety to a significantly greater extent than males. Regarding the perception of the last testing, the differences between males and females were found in three items (Q1, Q4, Q5) at the significance level of α < 0.05, while females again showed higher values. It was possible to state that females perceived testing more positively. The differences in perception were more pronounced in age groups compared to gender groups. The significant differences were observed in almost all cases and it was possible to confirm that younger groups of the population perceived testing more positively than older groups.

**Table 3:** COVID-19 testing perceptions–the first test and the last test in the classification of gender and age.

			**Q1**	**Q2**	**Q3**	**Q4**	**Q5**	**Q6**
**Gender**								
T1	Males	Mean	2.92	2.76	2.63	2.38	2.88	2.82
		Median	3.00	3.00	3.00	2.50	3.00	3.00
	Females	Mean	3.06	2.92	2.82	2.59	3.15	2.82
		Median	3.00	3.00	3.00	3.00	4.00	3.00
	Diff	Z	−1.43	−1.62	−2.22^**^	−2.63^***^	−2.85^***^	−0.31
T2	Males	Mean	2.61	2.41	2.35	2.17	2.75	2.95
		Median	3.00	2.00	2.00	2.00	3.00	3.00
	Females	Mean	2.83	2.57	2.47	2.42	3.01	2.92
		Median	3.00	3.00	2.50	2.00	3.00	3.00
	Diff	Z	−2.23^**^	−1.78^*^	−1.42	−3.08^***^	−2.76^***^	−0.60
**Year of birth**								
T1	<1980	Mean	2.53	2.36	2.27	2.10	2.59	2.92
	(>41 years)	Median	3.00	2.00	2.00	2.00	3.00	3.00
	1980–1989	Mean	2.71	2.59	2.48	2.20	2.78	2.84
	(32–41 years)	Median	3.00	3.00	3.00	2.00	3.00	3.00
	1990–1999	Mean	3.23	3.07	2.96	2.68	3.26	2.75
	(22–31 years)	Median	3.00	3.00	3.00	3.00	4.00	3.00
	2000+	Mean	3.27	3.16	3.06	2.87	3.30	2.92
	(<22 years)	Median	3.50	3.00	3.00	3.00	4.00	3.00
	Diff	H	50.93^†^	54.22^†^	55.36^†^	53.95^†^	46.14^†^	3.81^†^
T2	<1980	Mean	2.33	2.24	2.14	2.00	2.49	2.91
	(>41 years)	Median	2.00	2.00	2.00	2.00	3.00	3.00
	1980–1989	Mean	2.42	2.24	2.13	1.94	2.61	2.96
	(32–41 years)	Median	3.00	2.00	2.00	2.00	3.00	3.00
	1990–1999	Mean	2.91	2.62	2.53	2.45	3.07	2.93
	(22–31 years)	Median	3.00	3.00	3.00	2.00	3.00	3.00
	2000+	Mean	3.08	2.83	2.80	2.77	3.30	2.91
	(<22 years)	Median	3.00	3.00	3.00	3.00	4.00	3.00
	Diff	H	41.63^†^	24.83^†^	29.49^†^	46.26^†^	43.90^†^	0.24

*T1, first test; T2, last test; Diff, differences*;

* *p-value < 0.1*;

** *p-value < 0.05*;

*** *p-value < 0.01*;

† *p-value < 0.001*.

The following part is devoted to the assessment of the links between the overall perception of testing and gender-age characteristics. The overall perception was expressed as the sum of individual items (Q1–Q6) in both the first and the last testing. Using Cronbach's α, the reliability was 0.782 in the first testing and 0.789 in the last testing. This could be considered as acceptable values to support the sum of the individual items and the creation of a given overall perception characteristic for the first testing (Perc_test T1), as well as for the last testing (Perc_test T2). Basic descriptive characteristics for overall perceptions were also provided (Perc_test T1 = mean: 16.99, median: 18, minimum: 6.00, maximum: 24.00; Perc_test T2 = mean: 15.81, median: 16, minimum: 6.00, maximum: 24.00), while the last testing showed significantly lower perception values (Z = −11.50, *p*-value < 0.001). The suitability of the correspondence analysis was supported by Pearson's χ^2^ test, the results of which revealed significant differences in gender-age category for Perc_test T1 (χ^2^ = 75.63, *p*-value < 0.001), as well as for Perc_test T2 (χ^2^ = 69.51, p-value < 0.001). The correspondence analysis included overall perception values defined in quartiles from least positive perception (≤ Q1) to most positive perception (Q3+).

**Figure 2:**
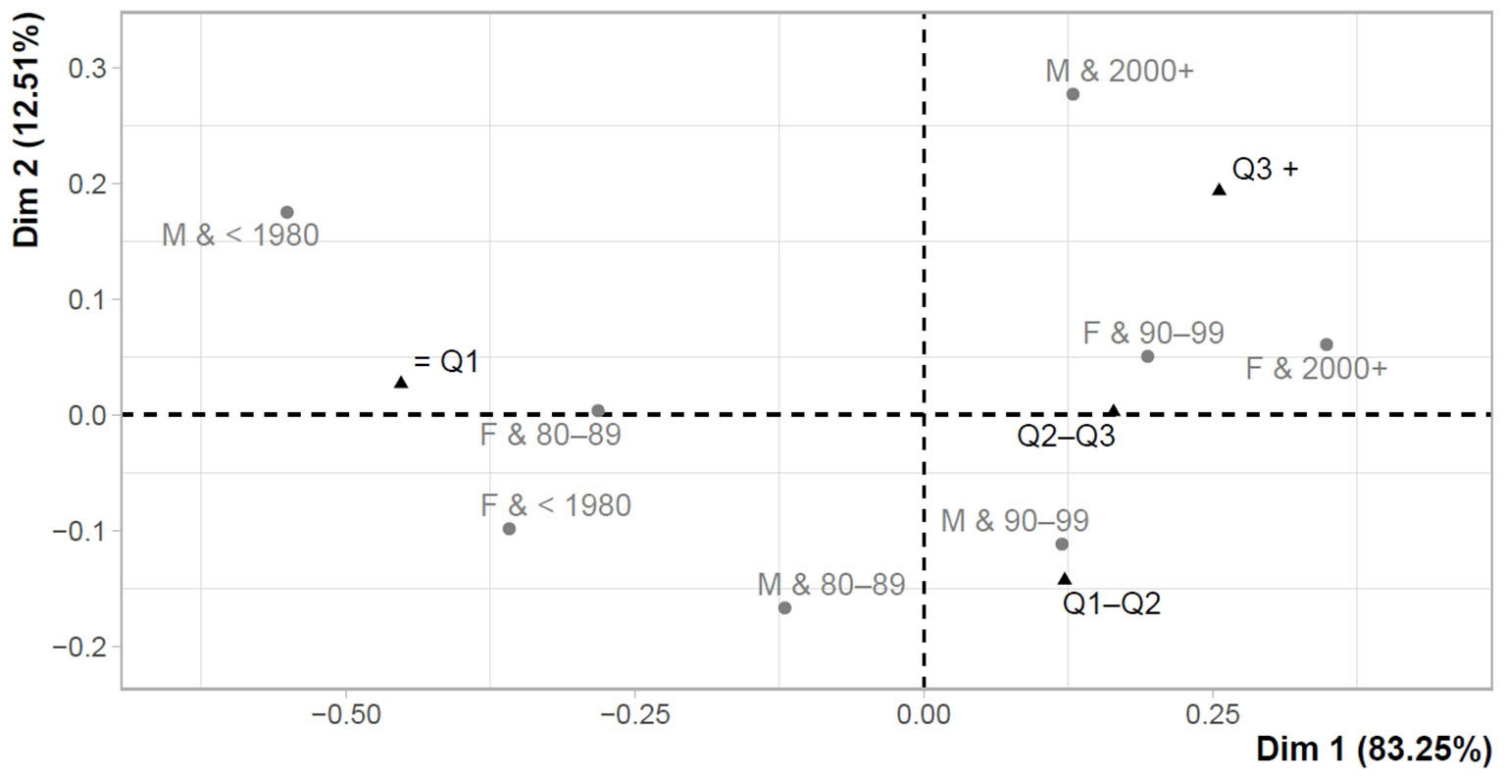
Correspondence map–Perc_test T1 and gender-age characteristics.

Figure 2 shows the links between the overall perception of the first testing (Perc_test T1) and gender-age characteristics. It was possible to observe relatively close links between the most positive perception of testing (Q3+) and younger groups of males and females (Males&2000+ (<22 years); Females&2000+ (<22 years); Females&1990–1999 (22–31 years)). These female groups were also closely concentrated to the second most positive perception of testing (Q2–Q3). The closest link with the least positive perception of the first testing was found in females aged 32–41 years (Females&1980–1989).

**Figure 3:**
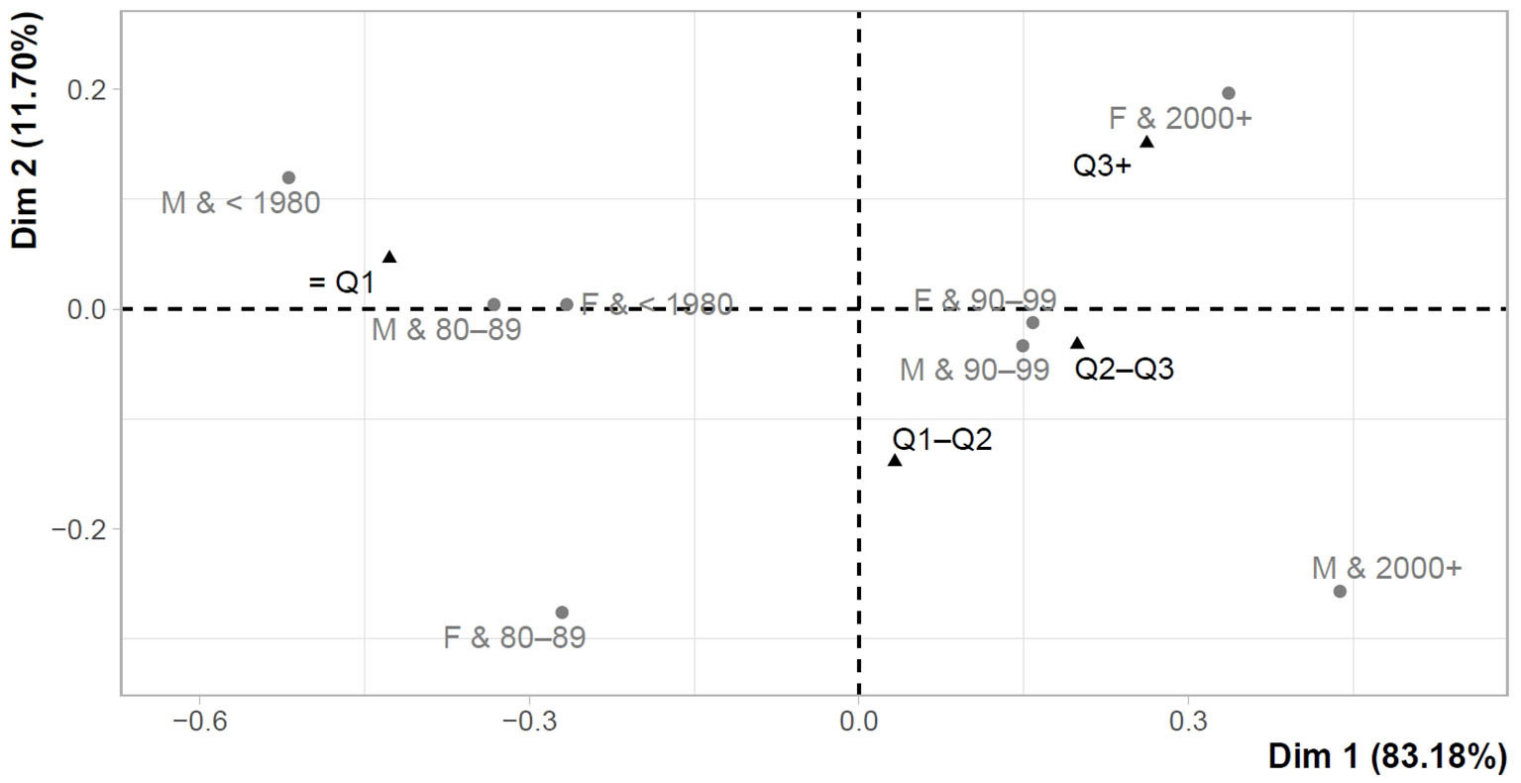
Correspondence map–Perc_test T2 and gender-age characteristics.

Figure 3 shows the links between the overall perception of the last testing (Perc_test T2) and gender-age characteristics. The close link was observed between the most positive perception of testing (Q3+) and the youngest females under the age of 22 years (Females&2000+). From the opposite point of view, the least positive perception of testing (≤ Q1) was characteristic of older males and females (Males&1980–1989 (32–41 years); Females& <1980 (>41 years); Males& <1980 (>41 years)).

### Perception of Doubts About the Accuracy of the COVID-19 Test Results

This part focuses on examining the perception of doubts about the accuracy of the COVID-19 test results. Table 4 shows the perception of doubts and it can be observed that there were significant differences between the first test and the last test without classification (ALL). Based on the higher mean value for the last test (T2), the last participation in testing was characterized by a higher level of doubts about the accuracy of the test results. Focusing on the time aspect of testing (less than a month, more than a month), there was no difference in the shorter time interval between tests, but a significant difference was evident in the longer time interval between tests. Similar results were found in the classification according to the number of tests. Thus, the significant differences in the perception of doubts between the first test and the last test were confirmed in the group with a larger number of tests. In the context of experience (in the form of the number of tests) and time (in the form of the time interval between tests), it was possible to conclude that the time aspect is an important element in perceiving doubts about test results.

**Table 4:** Perception of doubts about the accuracy of the COVID-19 test results–the first test and the last test in the classification of time between tests and the number of tests.

	**T1**					**T2**					**Diff**
	***n***	**Mean**	**Q1**	**Med**	**Q3**	***n***	**Mean**	**Q1**	**Med**	**Q3**	**T1–T2**
**All**	806	3.94	3	4	5	768	4.02	3	5	5	−2.53^†^
**Time interval between the first and the last test**											
Less than a month	114	4.05	3	5	5	112	3.88	3	5	5	−1.35
More than a month	659	3.91	3	4	5	655	4.04	3	5	5	−3.47^***^
**Number of tests**											
≤ 5	435	4.01	3	5	5	400	3.99	3	5	5	−0.17
6+	366	3.85	3	4	5	362	4.07	3	5	5	−4.01^†^

*T1, first test; T2, last test; Diff, differences*;

*** *p-value < 0.01*;

† *p-value < 0.001*.

Differences across gender and age groups were also assessed in the case of examining the perception of doubts about the accuracy of test results. Regarding the first participation in testing, the values of the central tendencies indicated some differences in the perception of doubts between males and females (Males = mean: 4.04, median: 5.00; Females = mean: 3.88, median: 4.00). The significance of these gender differences was supported (Z = −2.259; *p*-value = 0.024), with males showing a higher level of doubts about the accuracy of test results compared to females. Similar findings were revealed for the last participation in testing (Males = mean: 4.15, median = 5.00; Females = mean: 3.94, median: 5.00), and significant gender differences were also confirmed (Z = −2.421; *p*-value = 0.015). With a focus on the age aspect at the first participation (<1980 = mean: 3.86, median: 5.00; 1980–1989 = mean: 4.23, median: 5.00; 1990–1999 = mean: 3.92, median: 4.00; 2000+ = mean: 3.82, median: 4.00), it was also possible to observe significant differences (H = 9.762; *p*-value = 0.021). In the last testing (<1980 = mean: 3.88, median: 5.00; 1980–1989 = mean: 4.23, median: 5.00; 1990–1999 = mean: 4.06, median: 5.00; 2000+ = mean: 3.83, median: 4.00), the result indicated a significant difference between age groups, but only at the level of α < 0.1 (H = 7.32; *p*-value = 0.062).

**Figure 4:**
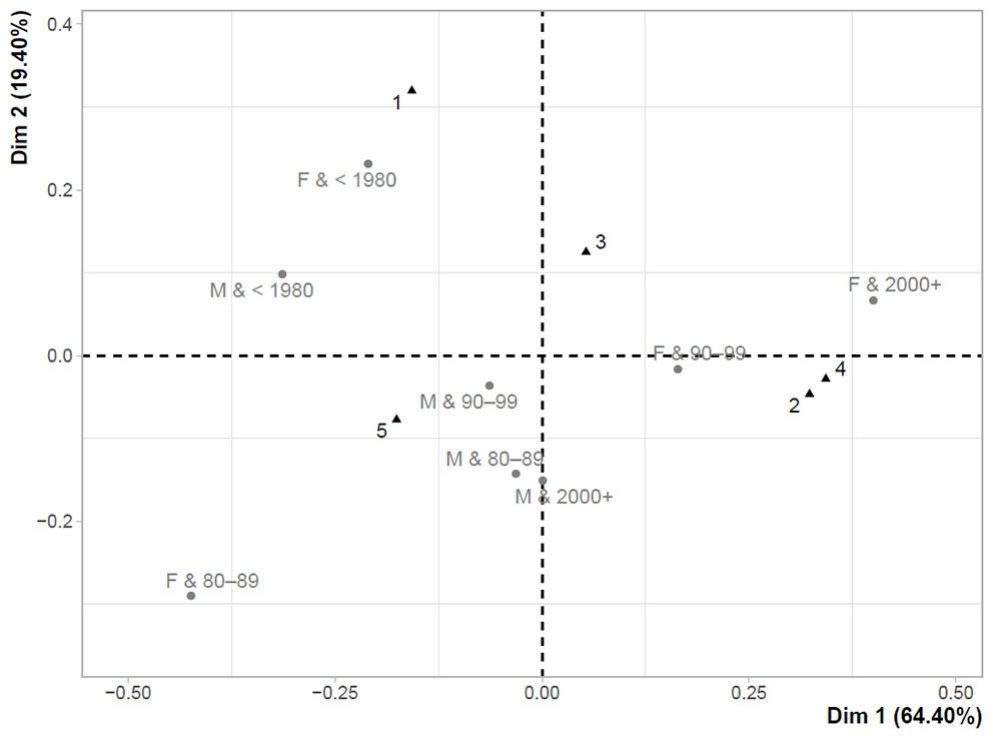
Correspondence map for T1–perception of doubts about the accuracy of test results and gender-age characteristics.

**Figure 5:**
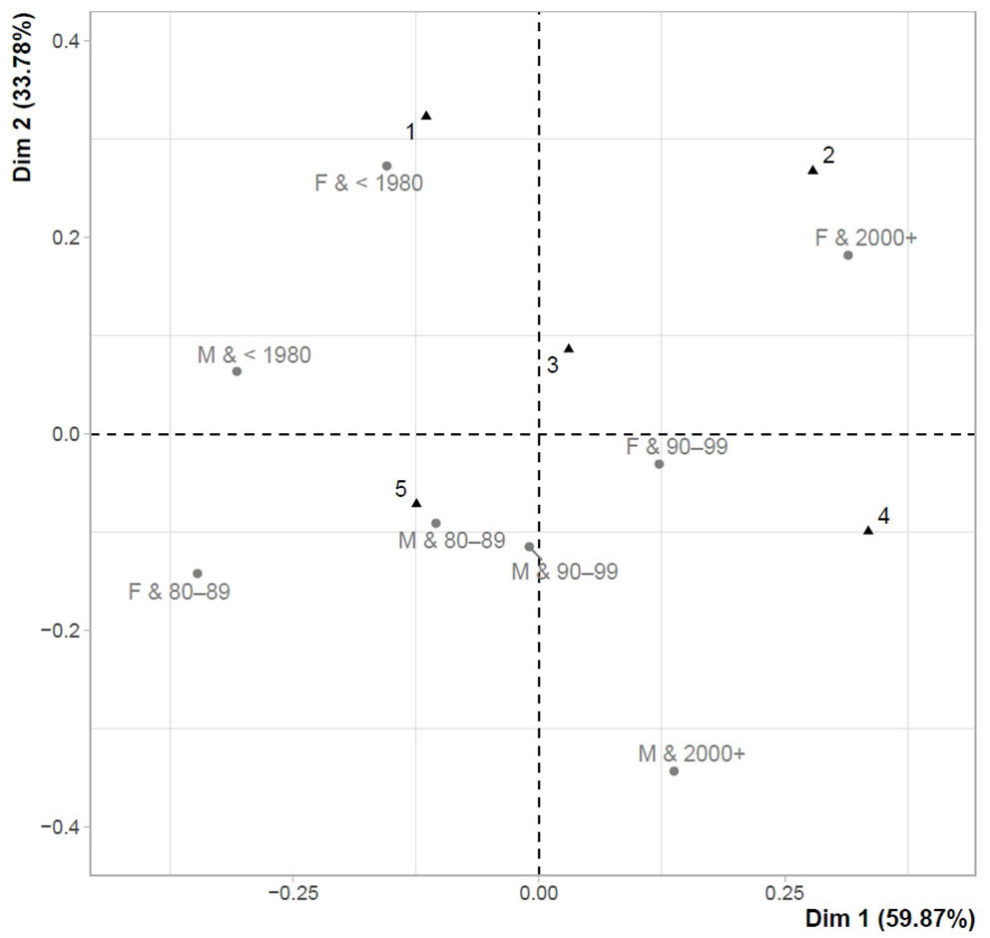
Correspondence map for T2–perception of doubts about the accuracy of test results and gender-age characteristics.

Figures 4, 5 show the maps of the correspondence analysis, which was conditioned by the significant value of Pearson's χ^2^ test in both examined cases (first testing (T1) = χ^2^: 61.79, *p*-value < 0.001; last testing (T2) = χ^2^: 43.29, *p*-value = 0.033). The maps present the links between the perception of doubts about the accuracy of test results and the characteristics of gender and age. The perception of doubts ranges from 1 to 5, with 5 indicating that a respondent was fully aware that the test result may not be correct. In this sense, a higher value indicated a higher level of doubts about the accuracy of the test results.

Figure 4 shows the links between the perception of doubts about the accuracy of test results and gender-age characteristics for the first participation in testing (T1). The closest link with the highest level of doubts (5) was found mainly in males (Males&1980–1989 (32–41 years); Males&1990–1999 (22–31 years); Males&2000+ (<22 years)), while older males (Males& <1980 (>41 years)) were slightly further from this point. Females over the age of 41 years (Females& <1980) were least aware of the fact that their test results may not have been correct and they were closely concentrated to the lowest level of doubts about the accuracy of the test results (1). Very similar results could be observed in the last testing (T2) shown in Figure 5.

### Behavior After Testing

At this point, it can be noted that people's behavior after COVID-19 testing poses a certain level of risk. A negative test result can encourage people to mobilize and meet others. False-negative people who do not know about their infection can be a threat, while the test result can convince them of their good health. The results revealed that 77.6% of respondents went home after the first testing, 15.7% of respondents met other people outside their household, while these respondents had information about a negative result of the COVID-19 test of people they met. The risk group consisted of 6.6% of respondents who met people outside their household without information about the test result. For people who did not go home after the first testing and they met someone, the most frequent reason was work, followed by a family reunion, entertainment, and the least frequent reason was sport. The findings from the last testing were similar, but slightly increased values appeared in the risk groups. It could be stated that 66.7% of respondents went home, 20.8% of respondents met other people with a negative test result, 7.8% of respondents met other people without information about the test result and 0.4% of respondents met other people with a positive test result. The most frequent reasons for the meeting were work, family and entertainment, while sport was the reason with the lowest frequency. In cases of meeting a positive person (3 observations, 0.4%), the reasons were a family reunion, work, but also entertainment.

## Discussion

Various countries used various tools and procedures to defeat the COVID-19 pandemic. The Slovak Republic is a country that has decided to take a courageous step, which has gained the attention of other countries as well. Mass testing of the entire population was chosen as the dominant part of the strategy to defeat the pandemic. This step was associated with different views and thus, it was possible to examine the attitudes and perceptions of testing across the Slovak population. In fact, the situation is perceived from the position of public leaders who make decisions, but also from the position of the society that bears individual political decisions (22). The COVID-19 pandemic has serious consequences for society, and therefore the leaders of national economies must be especially careful in choosing a successful strategy so as not to exacerbate social discomfort (23). In other words, the social aspect should not go sideways.

Some evidence suggests that asymptomatic testing is highly acceptable (24), especially in the explosive phase of an epidemic (25). However, the perception and motivation of such people to participate in testing should not be forgotten. There are many factors that can affect people's testing perceptions and their willingness to participate in testing. In this context, the results of this study highlighted the time aspect, testing experience, but also gender and age. According to Vandrevala et al. (26), closely following the news media was positively linked to willingness to be tested, while view on testing, knowledge and perceptions about COVID-19 predicted the willingness to test. In addition, stigma, trust in institutions, expectations regarding the integrity of tests (20) or other obstacles and concerns (21) could also play an important role in the willingness to participate in testing. All these aspects should be taken into account in the development of successful testing strategies and other strategies aimed at defeating the COVID-19 pandemic.

Regarding the results of this study, there were significant differences in perception between the first participation in testing and the last participation in testing. It was revealed that the level of internal motivation represented by the feeling that participation in testing is right, beneficial, helpful society, responsible to other people and evoking a sense of security, was significantly lower at the last participation in testing. Also, external incentives represented by government regulations and restrictions (if people did not participate) were higher in the last testing. From a social point of view, these results could not be considered positive. The differences in perception between the first test and the last test were more intense in the group with a longer time interval between tests. On this basis, it can be assumed that the time during which information on the pandemic or on testing (from different sources and with different relevance) could have an adverse effect on the perception of testing. Similar results were found in the groups classified according to the number of tests, while differences in perception between the first test and last test were confirmed in all but one case. The difference in perception was not confirmed only in the group with a lower number of tests in the case of external stimulation by restrictions. This result indicated that government regulations were more strongly perceived in the group that was tested more frequently.

When assessing differences in testing perceptions, some differences were evident in terms of gender, while in cases of personal sense of security or responsibility for people, a significantly higher rate of positive testing perception was found in females compared to males. In terms of age, it was revealed that the younger groups perceived their participation in testing more positively than the older groups. Similar results were identified by correspondence analysis. Thus, at the first participation in testing, a higher rate of positive perception was more closely linked to the younger population, and a lower rate of positive perception was identified in females aged 32–41 years. At the last participation in testing, the group of people with the least positive perception of testing was expanded to include older males as well as females.

Opportunities to improve testing perception are provided by home self-tests or pharmacist-provided testing services (27, 28), which allow comfortable use at the moment of need and without feeling compelled. Preprint research evidence showed that self-testing can provide similar results to professional testing, while antigen-detecting rapid diagnostic tests have proven to be relatively easy to perform by non-professionals (27). People's willingness to test is important, as testing is one of the crucial tools for controlling the COVID-19 pandemic (18, 19, 29). In this context, self-testing could positively motivate people to regularly and voluntarily monitor their health and to be responsible for the people with whom they are in close contact. In this way, the population could be effectively tested twice a week, which helps to reduce the risk of outbreaks (30, 31).

In population-wide testing, it should be borne in mind that rapid antigen tests can be inaccurate to some extent. Doubts about the accuracy of rapid antigen tests stem from their lower diagnostic sensitivity compared to RT-PCR (8–10, 12). From a social point of view, it is necessary to focus on false-positive and false-negative results. For false-positive individuals, this means strict isolation despite an incorrect result, and they could be falsely identified as non-infectious in terms of overcoming the disease after ten days. False-negative individuals could pose a health threat to society, as they could unknowingly transmit the infection to a community based on incorrect information. Thus, the spread of the virus may not be under control and all these facts could undermine public trust toward testing.

Analysis of the perception of doubts about the accuracy of the test result revealed that most people realized that their result may not be correct. A significant difference in this perception between the first test and the last test indicated that the level of doubts increased over time. The result can be considered in a positive context, as there is a presumption that if people realize that their negative result may be false, they will continue to behave with caution. They can avoid risky behavior. From a gender point of view, significantly higher doubts were measured in males at the first participation in testing, as well as in the last. In terms of age, a difference with a significance level of α < 0.05 was identified for the first testing. The lowest level of doubts appeared in the youngest population born in 2000 and later (<22 years), the highest level of doubts was measured in the population group born between 1980 and 1989 (32–41 years). The results of the correspondence analysis showed that the group with the lowest level of doubts included older females.

Last but not least, it should be noted that some people met other people after testing, despite the possible risks and restrictions. The most common reason for the meeting was work, followed by a family reunion, then entertainment, and the least frequent reason for the meeting was sport. Behavioral responses to mass asymptomatic testing are very important, as false-negative results may cause serious harm due to failure to detect infection (32). A negative result may lead to a false reassurance of good health and a consequent behavior change in society. The potential increase in risk behavior after a negative result was also shown by the evidence from Liverpool, which revealed that 23% of those who tested negative would be more likely to go out for a walk or exercise, 17% more likely to go to the shops, 9% more likely to visit friends and family, and 7% more likely to go to work (33). In this study, 22.4% of respondents met other people after their first test and 33.3% of respondents met other people after their last test, indicating an increased risk.

It is also necessary to take into account the understanding of medical test results by people without a medical background (34), while health literacy plays an important role in the general population (35). In any case, public health representatives should provide the public with clear messaging on the use and limitations of rapid antigen testing. People should be aware that a negative test result does not rule out the possibility of infection today or in the days to come. People need to be educated to understand the meaning of positive or negative results of a rapid antigen test, and they should not forget about other important measures such as physical distance, masking, hand hygiene, but also vaccination (18). The combination of social distance and testing is still an effective strategy in reducing the burden of disease (36). Increasing access and education on non-pharmaceutical interventions could reduce viral spread (37), but emphasis should also be placed on the social aspect, positively motivating people, improving their attitudes, but also their trust. There are many social and cultural factors that shape perceptions and responses to risk messaging, while outrage factors, danger, immediacy, uncertainty, familiarity, personal control, scientific uncertainty, and trust in institutions and media appear to be key (38, 39). How detailed the messages are communicated also plays an important role in complying with the measures (40). In this way, hospitals could be protected from the large number of infected patients they have faced.

In these critical situations, the key aspect is the hospital management (41), but also health care expenditure to provide adequate services required by the circumstances (42). Any crisis is costly and therefore sensible and effective measures need to be taken to overcome the COVID-19 pandemic (43, 44). It is important for policy-makers to realize that the decisions they make at a national-wide level affect society, but also economic life. The prognosis shows negative events which will decrease slowly (45). One possible strategy is mass testing, which has been highlighted in other studies whose authors have recommended involving the population in frequent testing along with appropriate isolation (46–49). On the other hand, these studies did not take into account society as such, but also the attitudes, perceptions, behaviors and willingness of individuals. The presented study enriches the current state of knowledge by clarifying the social aspect of the COVID-19 pandemic, which is important in addition to the health and economic aspects. This study encourages the proactive approach of policy makers and the implementation of effective and evidence-based strategies that take into account the social perspective. A valuable platform of research results can help this effort.

### Limitations and Future Directions

This research did not avoid the limitations to which the disproportionate nature of the sample could be included. Thus, there was a higher proportion of females and the social status of students (younger respondents). Due to the use of the online survey, there were some deviations from the country population. Therefore, the findings should be generalized with caution. However, this limitation need not be considered disruptive to the results and value of knowledge. Another possible limitation is the fact that the research focused exclusively on the Slovak population and therefore did not provide more universal generalizations. The questionnaire did not contain items identifying other deeper attitudes of the respondents. Therefore, it is considered an inspiring idea for future research to clarify the problem in more detail. At the same time, future research should take into account the source of information on testing available to the population, as well as personal perceptions of health, self-care and self-monitoring, which could be other factors in testing attitudes.

## Conclusion

The objective of the presented research was to examine the perception of COVID-19 testing in the Slovak population. In the Slovak Republic, a testing strategy has been implemented at the level of the entire population, which does not occur in other countries. This underlines the originality of the study, while the novelty is a closer look behind the testing scene from the position of the society as such. Evidence has shown that the rate of positive perception of testing has decreased and the level of doubts has increased. At the same time, there were significant differences in terms of gender and age of the Slovak population, but also in terms of testing experience and time. People's willingness to test plays an important role in managing the COVID-19 pandemic, with public health leaders having a significant impact. People should be positively encouraged to participate in testing that could help overcome the COVID-19 pandemic.

Based on the results, it can be concluded that the frequency of testing and its requirements need to be approached very carefully over time, as it is likely that the population will experience a deterioration in their positive perception. People should understand the need and importance of testing in order to improve the situation and help society, but this is not possible without clear information and a meaningful regime from public leaders. Otherwise, negative feelings and doubts over time may dominate the perception of the entire population, posing a risk of outbreaks. It is very important to focus on the positive perceptions and attitudes of the population, which are key to the successful management of the pandemic. The focus is not to eliminate individual risk, but to reduce risk at the population level. The major recommendations include clear and timely government communication, positive motivation, trust building, fear elimination and health education. Last but not least, testing in outbreaks with a high viral load, in people with early symptoms, as well as in regions, population groups or places with higher incidence and risk, seems to be a more appropriate strategy. The purpose of such testing could be more acceptable to the population in terms of their perceptions and attitudes.

## Data Availability Statement

The raw data supporting the conclusions of this article will be made available by the authors, without undue reservation.

## Ethics Statement

The studies involving human participants were reviewed and approved by the Ethics Committee of the Clinical Trials Services, USP TECHNICOM, Technical University of Košice, Slovakia (Ref. 02/03/2021 IG Bioinformatics). All participants were informed of the study purpose and signed an informed consent regarding participation in the study. This study was conducted with respect to the seventh revision of the World Medical Association Declaration of Helsinki. The patients/participants provided their written informed consent to participate in this study.

## Author Contributions

BG: conceptualization, writing—original draft preparation, writing—review and editing, visualization, supervision, project administration, and funding acquisition. VI: conceptualization, investigation, resources, writing—original draft preparation, writing—review and editing, visualization, and supervision. MR: conceptualization, methodology, software, data curation, formal analysis, investigation, writing—original draft preparation, and writing—review and editing. ZC: investigation, writing—review and editing, visualization, supervision, project administration, and funding acquisition. TM: resources, writing—review and editing, visualization, supervision, project administration, and funding acquisition. All authors contributed to manuscript revision, read, and approved the submitted version.

## Funding

This research was funded by the Scientific Grant Agency of the Ministry of Education, Science, Research, and Sport of the Slovak Republic and the Slovak Academy Sciences as part of the research project VEGA 1/0797/20: Quantification of Environmental Burden Impacts of the Slovak Regions on Health, Social and Economic System of the Slovak Republic. This research was supported by the Slovak Research and Development Agency under the contract No. APVV-17-0360: Multidimensional analysis of significant determinants of public procurement efficiency with emphasis on the application of Health Technology Assessment in the procurement preparation phase.

## Conflict of Interest

The authors declare that the research was conducted in the absence of any commercial or financial relationships that could be construed as a potential conflict of interest.

## Publisher's Note

All claims expressed in this article are solely those of the authors and do not necessarily represent those of their affiliated organizations, or those of the publisher, the editors and the reviewers. Any product that may be evaluated in this article, or claim that may be made by its manufacturer, is not guaranteed or endorsed by the publisher.
