# Supplementary figures and images for: Perception of COVID-19 Testing in the Entire Population
Source: Front Public Health. 2022 Feb 10;10:757065. doi: 10.3389/fpubh.2022.757065 (PMC8877808; doi:10.3389/fpubh.2022.757065)

Supplementary Material

**
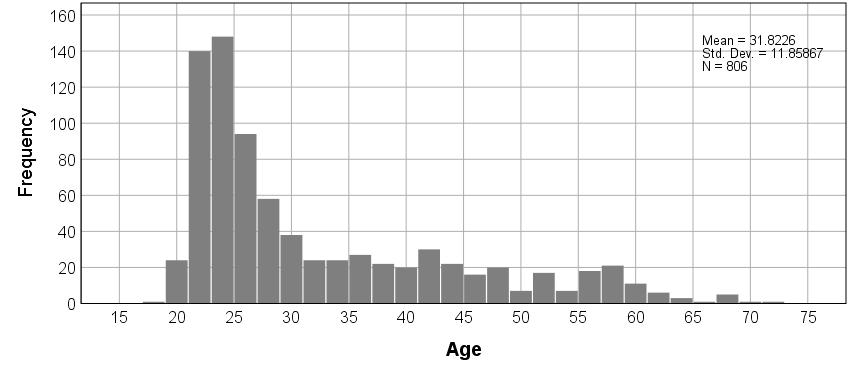
**

**Supplementary Figure 1.** Histogram of individual age categories

Supplement: Supplementary file 1 [file Table_1.DOCX]
